# Prevalence and Severity Distribution of Type 2 Inflammation-Related Comorbidities Among Patients with Asthma, Chronic Rhinosinusitis with Nasal Polyps, and Atopic Dermatitis

**DOI:** 10.1007/s00408-023-00603-z

**Published:** 2023-02-20

**Authors:** Asif H. Khan, Imène Gouia, Siddhesh Kamat, Robert Johnson, Mark Small, James Siddall

**Affiliations:** 1 Sanofi, Chilly-Mazarin, France; 2 Regeneron Pharmaceuticals, Inc., Tarrytown, NY USA; 3 Sanofi, Bridgewater, NJ USA; 4 Adelphi Real World, Bollington, UK

**Keywords:** Allergic rhinitis, Asthma, Atopic dermatitis, Chronic rhinosinusitis with nasal polyps, Real-world clinical practice, Type 2 inflammation-related comorbidities

## Abstract

This observational study assessed the prevalence of co-existing type 2 inflammatory conditions [T2Cs; asthma, atopic dermatitis (AD), allergic rhinitis, and chronic rhinosinusitis with nasal polyps (CRSwNP)] in patients with moderate-to-severe (M/S) type 2 asthma, M/S CRSwNP, or M/S AD, in the real-world setting. Data from 761 physicians in the US and EUR5 were sourced from Adelphi Disease-Specific Programmes covering patients with M/S asthma (*n* = 899), M/S CRSwNP (*n* = 683), and M/S AD (*n* = 1497). At least one T2C was identified in 66%, 69%, and 46% of M/S asthma, M/S CRSwNP, and M/S AD cohorts, respectively, and 24%, 36% and 16% had at least two T2Cs; trends were similar in the US and EUR5. In patients with M/S asthma or M/S CRSwNP, T2Cs commonly presented as mild or moderate. The comorbidity burden suggests that an integrated treatment approach is warranted to address underlying type 2 inflammation in patients with M/S type 2 diseases.

**Supplementary Information:**

The online version contains supplementary material available at 10.1007/s00408-023-00603-z.

## Introduction

The overexpression of type 2 inflammatory pathways, involving activation of T helper type 2 cells and type 2 innate lymphoid cells [1], significantly contributes to the pathogenesis of asthma, allergic rhinitis (AR), chronic rhinosinusitis with nasal polyps (CRSwNP), and atopic dermatitis (AD) [1–3]. This common underlying pathology leads to the frequent co-existence of these conditions with varying levels of severity and places a high burden on affected individuals and their families [4–6]. The contribution of genetic mutations has been assessed in several genomic wide association studies, and multiple loci have been associated with more than one atopic condition including (but not limited to) 5q22, 6p21, 11q13, and 16p13; findings from these studies have been neatly summarized in two recent review articles [7, 8]. Taken together, these findings suggest that certain genetic anomalies may predispose individuals not only to manifestation of these allergic conditions, but to their comorbid co-existence.

Quantifying the overlap of co-existing type 2 inflammatory diseases (T2Cs) in asthma, CRSwNP, and AD will allow a better understanding of the associated prevalence and disease burden. Patients with comorbid diseases are more refractory to treatment for their primary disease, and such data will help provide insight for clinicians regarding disease overlap. Limited data reporting on prevalence and severity of T2Cs among patients with moderate-to-severe (M/S) asthma, M/S CRSwNP, and M/S AD currently exists. In this study, prevalence of common T2Cs was evaluated in patients with M/S asthma, M/S CRSwNP, or M/S AD. The severity of T2Cs was also reported in patients with M/S asthma and patients with M/S CRSwNP.

## Methods

### Study Design

This was an observational, real-world study based on country-specific, point-in-time survey data collected from January 2017 through February 2020 from physicians practicing in the United States (US) and in five European countries (EUR5: France, Germany, Italy, Spain, United Kingdom).

### Data Sources

Real-world data were sourced from three Adelphi Disease-Specific Programmes (DSPs) covering asthma, CRSwNP, and AD. DSPs are cross-sectional, country-specific surveys of physicians and patients in routine clinical practice [9]. A sample of US and EUR5 primary care physicians and specialists (i.e., respiratory, ear, nose, and throat physicians, chest physicians, pulmonologists, allergists, and dermatologists) was contacted and asked to review medical records of patients with a physician-confirmed primary diagnosis of asthma, M/S CRSwNP, or M/S AD. Physicians who saw ≥ 3 patients per week and were responsible for treatment completed Patient Report Forms for consecutive patients (≥ 18 years) with one of the three primary diagnoses of interest. The severity of T2Cs was reported by physicians in the M/S asthma and M/S CRSwNP datasets.

### Inclusion Criteria

To be included in the analyses, patients with M/S type 2 asthma needed to have persistent use (≥ 3 months) of medium-to-high dose inhaled corticosteroid/long-acting beta-2 agonist *and* ≥ 1 exacerbation, with either blood eosinophil count ≥ 150 cells/μL *or* intermediate/high fractional exhaled nitric oxide (based on physician judgement); *or* alternatively were included if they had persistent (≥ 3 months) maintenance oral corticosteroid (OCS) dependence. Patients with M/S CRSwNP were required to have maintenance or acute OCS in their treatment history *or* ≥ 1 prior sinus surgery for CRSwNP *and* M/S “nasal blockage” in the past 2 weeks. Patients with M/S AD were required to have received topical therapy (corticosteroid of any strength, crisaborole, and/or a calcineurin inhibitor) *and* either had disease described by their physician as changeable, deteriorating slowly, deteriorating rapidly *or* were suitable candidates for a systemic therapy (immunosuppressants or injectable corticosteroids or biologics) according to their physician; *or* alternatively were currently receiving or had previously received a systemic therapy for AD.

### T2Cs Evaluated

Common T2Cs evaluated for patients with M/S asthma, M/S CRSwNP, or M/S AD were AR (evaluated in all three cohorts), asthma (evaluated in M/S CRSwNP and M/S AD cohorts), AD (evaluated in M/S asthma and M/S CRSwNP cohorts), and CRSwNP (evaluated in M/S asthma and M/S AD cohorts).

### Analyses

Descriptive analyses were conducted to determine patient demographics and prevalence of T2Cs among patients with M/S asthma, M/S CRSwNP, or M/S AD. Physician-assessed distribution of severity of T2Cs among patients with M/S asthma and M/S CRSwNP was also evaluated.

## Results

### Patient Demographics

Overall, 761 physicians from the US and EUR5 identified 899 patients with M/S asthma, 683 with M/S CRSwNP, and 1497 with M/S AD who met the study inclusion criteria. Patient demographics are summarized by disease cohort in Online Resource 1.

### Prevalence of T2Cs

Overall, at least one T2C was identified in 66%, 69%, and 46% of patients with M/S asthma, M/S CRSwNP, and M/S AD, respectively. Similar proportions were reported in the US (71%, 82%, and 51%) and EUR5 (65%, 65%, and 45%). Among patients with a primary diagnosis of M/S asthma, 3% had all three of the common T2Cs, 21% had two T2Cs, and 43% had one T2C. Similar proportions were reported in the US (1%, 17%, and 54%) and EUR5 (3%, 21% and 40%). For patients with a primary diagnosis of M/S CRSwNP, 4%, 32%, and 34% had three, two, and one T2Cs, respectively, with similar proportions reported in the US (2%, 49%, and 32%) and EUR5 (5%, 27%, and 34%). Among patients with a primary diagnosis of M/S AD, the respective proportions were 1%, 15%, and 30%. Similar proportions were reported in the US (1%, 22%, and 28%) and EUR5 (0%, 13%, and 31%).

**Fig. 1:**
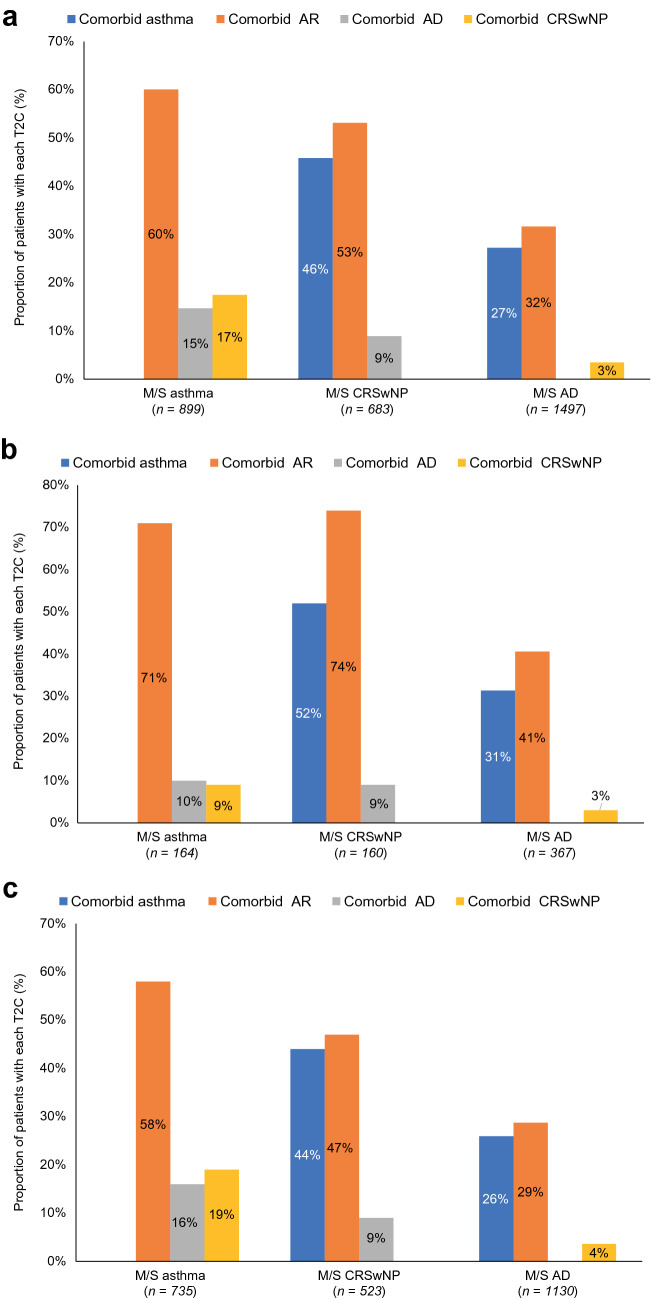
Proportions of patients in each disease cohort with T2Cs: **a** overall; **b** US; **c** EUR5. *AD* atopic dermatitis, *AR* allergic rhinitis, *CRSwNP* chronic rhinosinusitis with nasal polyps, *EUR5* France, Germany, Italy, Spain, United Kingdom, *M/S* moderate-to-severe, *T2C* co-existing type 2 inflammatory disease, *US* United States

Among patients in the M/S asthma cohort, there was a high prevalence (60%) of comorbid AR, while comorbid AD and comorbid CRSwNP occurred in 15% and 17% of cases (Fig. 1); in the US, the corresponding proportions reported were 71%, 10%, and 9%, respectively, and those for EUR5 were 58%, 16% and 19%, respectively. Prevalence of T2Cs in patients with M/S asthma across individual EUR5 countries ranged from 50 to 76% for comorbid AR, 13–24% for comorbid AD, and 12–31% for comorbid CRSwNP (Online Resource 2). Among patients in the M/S CRSwNP cohort, almost half had comorbid asthma (46%), and just over half had comorbid AR (53%); only 9% had comorbid AD (Fig. 1). The corresponding proportions in the US were 52%, 74%, and 9%, and in the EUR5 were 44%, 47%, and 9%. Prevalence of T2Cs in patients with M/S CRSwNP across individual EUR5 countries ranged from 31 to 55% for comorbid asthma, 38–55% for comorbid AR, and 7–11% for comorbid AD (Online Resource 2). Among patients in the M/S AD cohort, 27% and 32% had comorbid asthma and AR, respectively, and very few (3%) patients with M/S AD had comorbid CRSwNP (Fig. 1). The corresponding proportions in the US were 31%, 41%, and 3%, and in the EUR5 were 26%, 29%, and 4%.

### Severity of T2Cs

**Fig. 2:**
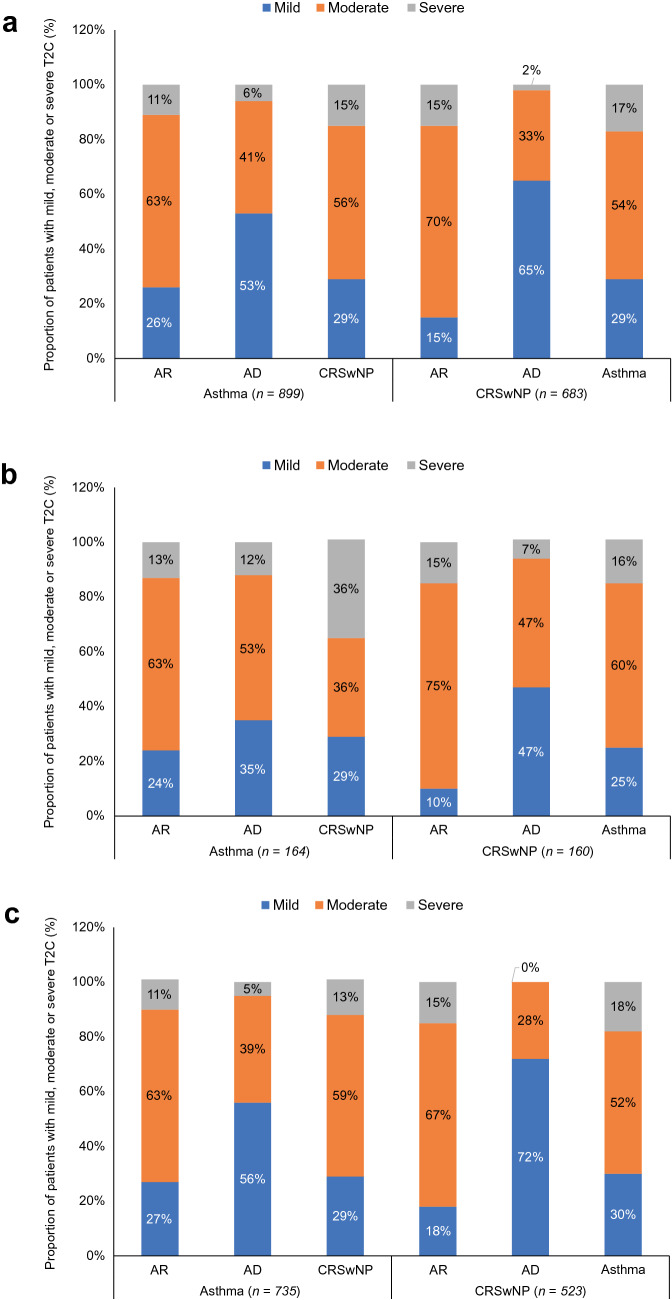
Physician-reported severity of T2Cs among patients with M/S asthma or M/S CRSwNP: **a** overall; **b** US; **c** EUR5. *AD* atopic dermatitis, *AR* allergic rhinitis, *CRSwNP* chronic rhinosinusitis with nasal polyps, *EUR5* France, Germany, Italy, Spain, United Kingdom, *M/S* moderate-to-severe, *T2C* co-existing type 2 inflammatory disease

**Fig. 3:**
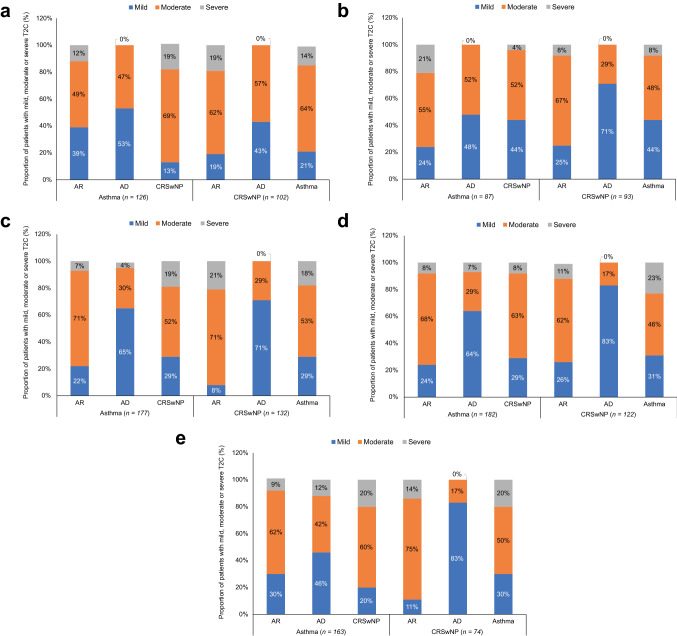
Physician-reported severity of T2Cs among patients with M/S asthma or M/S CRSwNP in EUR5 countries: **a** France; **b** Germany; **c** Italy; **d** Spain; **e** United Kingdom. *AD* atopic dermatitis, *AR* allergic rhinitis, *CRSwNP* chronic rhinosinusitis with nasal polyps, *EUR5* France, Germany, Italy, Spain, United Kingdom, *M/S* moderate-to-severe, *T2C* co-existing type 2 inflammatory disease

The distribution of physician-rated severity of T2Cs among patients with M/S asthma or M/S CRSwNP is shown in Fig. 2. Mild or moderate comorbid AR was identified in 26% and 63% of the M/S asthma cohort (US: 24% and 63%; EUR5: 27% and 63%), and in 15% and 70% of the M/S CRSwNP cohort (US: 10% and 75%; EUR5: 18% and 67%). Mild or moderate comorbid AD was reported in 53% and 41% of patients in the M/S asthma cohort (US: 35% and 53%; EUR5: 56% and 39%), and in 65% and 33% in the M/S CRSwNP cohort (US: 47% for both; EUR5: 72% and 28%). Mild or moderate comorbid CRSwNP was reported in 29% and 56% of patients in the M/S asthma cohort (US: 29% and 36%; EUR5: 29% and 59%). Mild or moderate comorbid asthma was reported in 29% and 54% of patients in the M/S CRSwNP cohort (US: 25% and 60%; EUR5: 30% and 52%); results were similar in individual EUR5 countries (Fig. 3).

## Discussion

The presence of co-existing disease has been shown to increase the burden of the primary disease in terms of clinical, health-related quality of life (HRQoL), and economic burden [6, 10, 11]. M/S type 2 diseases, including M/S asthma, M/S CRSwNP, and M/S AD, have been associated with a substantial burden of illness that negatively impacts patient HRQoL, and this is further exacerbated in patients with T2Cs and those with severe disease [6, 10, 12, 13]. Furthermore, although the co-existing diseases are mostly mild to moderate in severity, these patients are often refractory to treatment for their primary disease and are more difficult to effectively manage.

In this study, a high proportion (~ 50–70%) of patients with M/S asthma, M/S CRSwNP, or M/S AD presented with at least one T2C, and many patients had at least two T2Cs. In patients with M/S asthma or M/S CRSwNP, T2Cs commonly presented in mild or moderate forms and less frequently in severe forms; these trends were consistent within US and EUR5 cohorts. These findings are consistent with previous observations in patients with M/S type 2 diseases. Prevalence of comorbid asthma has reportedly been as high as 65% in a study assessing 40 consecutive patients with CRSwNP from a single Danish center [14]. Our prevalence observations of 46% are in line with a previous US single-center study reporting that 48% of patients with CRSwNP had comorbid asthma [15]. These estimates are clearly far higher than the 8.5% prevalence of asthma in the general US population [16]. Comorbid CRSwNP has been reported in 43% of patients with severe asthma in Italy [17]; this is considerably higher than the rate of CRSwNP comorbidity seen in patients with asthma in our Italian sample (18%), although it should be noted that our results also encompass patients with moderate asthma. The co-existence of asthma and rhinitis is also widely acknowledged, with some studies reporting rhinitis in as many as 75% of asthmatic patients [18], and AD is known to be a major risk factor for asthma and/or AR, with up to 70% of patients with severe AD reportedly developing asthma [18]. It should be noted that this study was an analysis of medical records of patients who were seeking care with a healthcare professional; therefore, this sample may not be representative of the general population in the US and/or EU.

The high comorbidity burden highlighted in this study, in terms of number and severity of T2Cs, suggests the importance of identifying patients with type 2 inflammation. The high prevalence of co-existing diseases in these patients highlights the importance of assessing comorbidities as part of routine care and the need for an integrated, multidisciplinary treatment approach to address underlying type 2 inflammation, and to improve HRQoL and overall outcomes in patients with type 2 diseases.

## Supplementary Information

Below is the link to the electronic supplementary material.

Supplementary file1 (DOCX 397 kb)

## Data Availability

Data may be made available by the corresponding author upon reasonable request.
