# Supplementary material for: Prevalence and Severity Distribution of Type 2 Inflammation-Related Comorbidities Among Patients with Asthma, Chronic Rhinosinusitis with Nasal Polyps, and Atopic Dermatitis
Source: Lung. 2023 Feb 20;201(1):57–63. doi: 10.1007/s00408-023-00603-z (PMC9968259; doi:10.1007/s00408-023-00603-z)
Supplement: Supplementary file 1 — Supplementary file1 (DOCX 397 kb) [file 408_2023_603_MOESM1_ESM.docx]

**Supplementary InformatioN**

**Prevalence and Severity Distribution of Type 2 Inflammation-Related Comorbidities Among Patients with Asthma, Chronic Rhinosinusitis with Nasal Polyps, and Atopic Dermatitis**

Asif H. Khan^1^, Imène Gouia^1^, Siddhesh Kamat^2^, Robert Johnson^3^, Mark Small^4^, James Siddall^4^

^1^Sanofi, Chilly-Mazarin, France; ^2^Regeneron Pharmaceuticals, Inc., Tarrytown, New York, USA; ^3^Sanofi, Bridgewater, New Jersey, USA; ^4^Adelphi Real World, Bollington, UK

**Corresponding author:** Asif H. Khan; [asif.khan@sanofi.com](mailto:asif.khan@sanofi.com)

**Online Resource 1.** Patient demographics

|  | **Overall**  **(n=3079)** | | | **EUR5**  **(n=2388)** | | | **US**  **(n=690)** | | |
| --- | --- | --- | --- | --- | --- | --- | --- | --- | --- |
|  | **Primary M/S asthma**  **(*n=899*)** | **Primary M/S CRSwNP**  **(*n=683*)** | **Primary M/S  AD**  **(*n=1497*)** | **Primary M/S asthma**  **(*n=735*)** | **Primary M/S CRSwNP**  **(*n=523*)** | **Primary M/S  AD**  **(*n=1130*)** | **Primary M/S asthma**  **(*n=164* )** | **Primary M/S CRSwNP**  **(*n=160*)** | **Primary M/S  AD**  **(*n=367*)** |
| **Age, mean (SD)** | 46.6 (15.4) | 47.2 (14.7) | 38.3 (15.1) | 46.3 (15.6) | 47.4 (14.2) | 37.7 (14.7) | 48.4 (14.7) | 46.5 (16.0) | 39.9 (16.3) |
| **Female, *n* (%)** | 544 (60.5) | 254 (37.2) | 751 (50.0) | 299 (39.3) | 193 (36.9) | 564 (49.9) | 66 (40.2) | 61 (38.1) | 187 (51.0) |
| **Race, *n* (%)** |  |  |  |  |  |  |  |  |  |
| White | 775 (86.2) | 588 (86.1) | 1262 (84.3) | 672 (91.4) | 479 (91.6) | 1000 (89.0) | 103 (62.8) | 109 (68.1) | 262 (71.8) |
| Black/African–American | 41 (4.6) | 24 (3.5) | 56 (3.7) | 9 (1.2) | – | 19 (1.7) | 32 (19.5) | 24 (15.0) | 37 (10.1) |
| Asian | 33 (3.7) | 0 | 95 (6.3) | 21 (2.9) | – | 60 (5.3) | 12 (7.3) | – | 35 (9.6) |
| Other | 50 (5.6) | 71 (10.4) | 77 (5.1) | 33 (4.5) | 44 (8.4) | 45 (4.0) | 17 (10.4) | 27 (16.9) | 32 (8.8) |
| **Employment status, *n* (%)** |  |  |  |  |  |  |  |  |  |
| Working full-time | 400 (44.5) | 412 (60.3) | 774 (52.0) | 301 (41.0) | 318 (60.8) | 557 (49.3) | 99 (60.4) | 94 (58.8) | 217 (59.1) |
| Working part-time | 109 (12.1) | 46 (6.7) | 125 (8.0) | 88 (12.0) | 31 (5.9) | 92 (8.1) | 21 (12.8) | 15 (9.4) | 33 (9.0) |
| Unemployed | 49 (5.5) | 34 (5.0) | 72 (5.0) | 40 (5.4) | 27 (5.2) | 67 (5.9) | 9 (5.5) | 7 (4.4) | 5 (1.4) |
| Retired | 125 (13.9) | 89 (13.0) | 101 (7.0) | 108 (14.7) | 70 (13.4) | 72 (6.4) | 17 (10.4) | 19 (11.9) | 29 (7.9) |
| Other | 216 (24.0) | 102 (14.9) | 425 (28.4) | 198 (26.9) | 77 (14.7) | 342 (30.3) | 18 (11.0) | 25 (15.6) | 83 (22.6) |

AD, atopic dermatitis; CRSwNP, chronic rhinosinusitis with nasal polyps; M/S, moderate-to-severe; SD, standard deviation

EUR5: France, Germany, Italy, Spain, United Kingdom.

**Online Resource 2.** Proportions of patients in each disease cohort with T2Cs in EUR5 countries: (**a**) France; (**b**) Germany; (**c**) Italy; (**d**) Spain; (**e**) United Kingdom

| **a** | 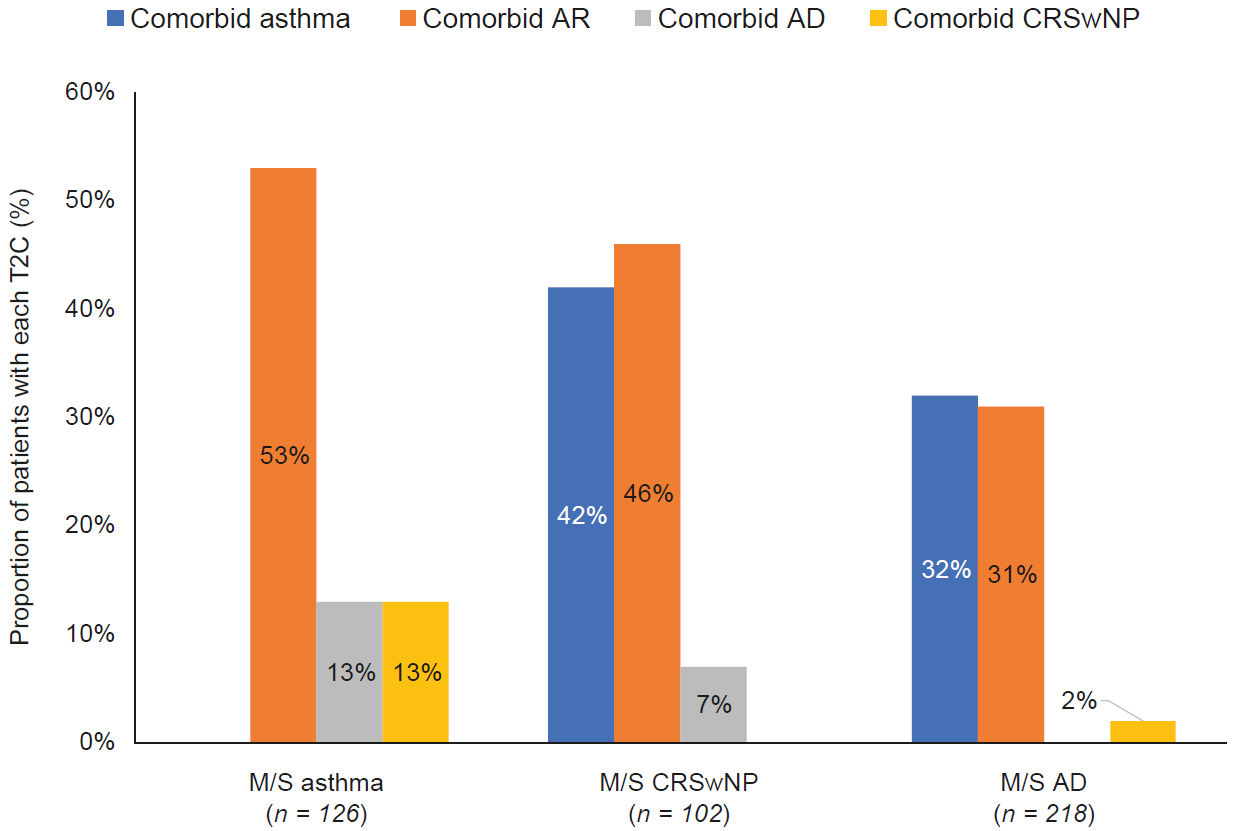 |
| --- | --- |
| **b** | 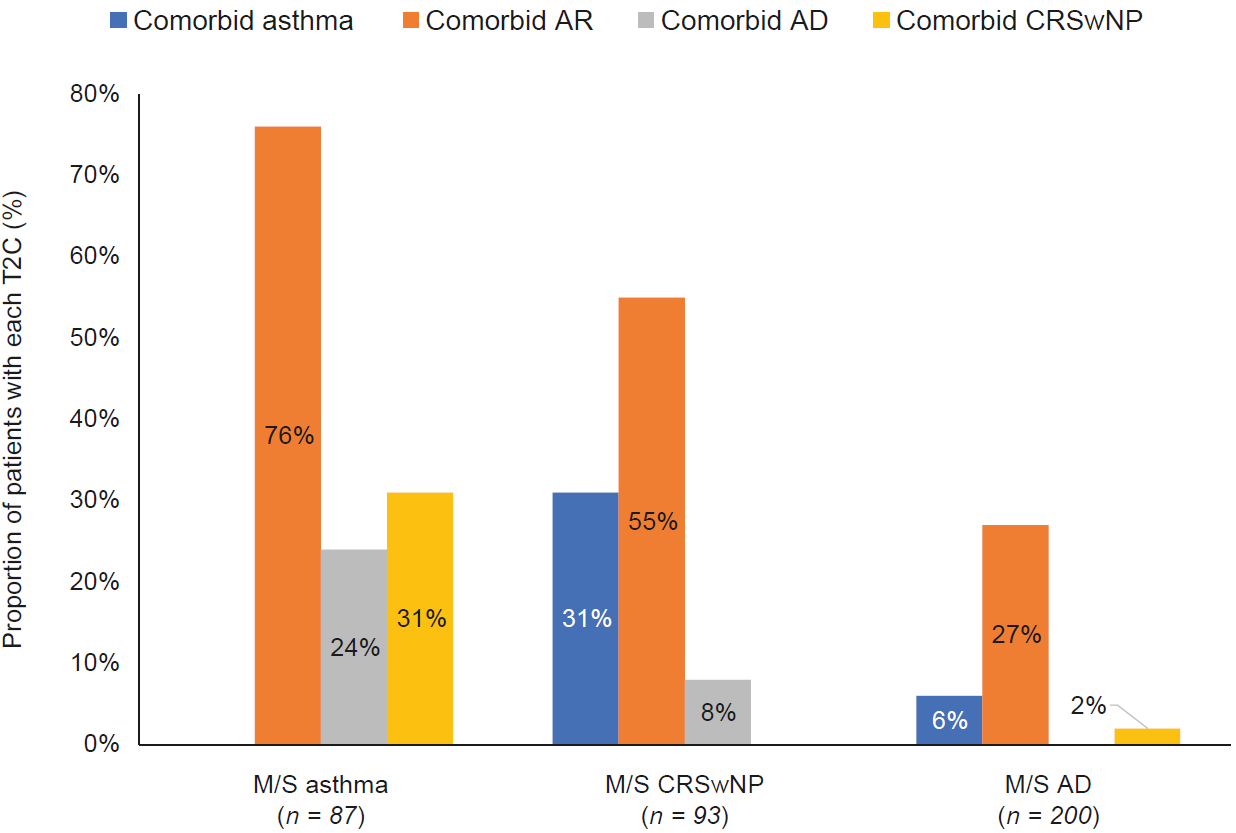 |
| **c** | 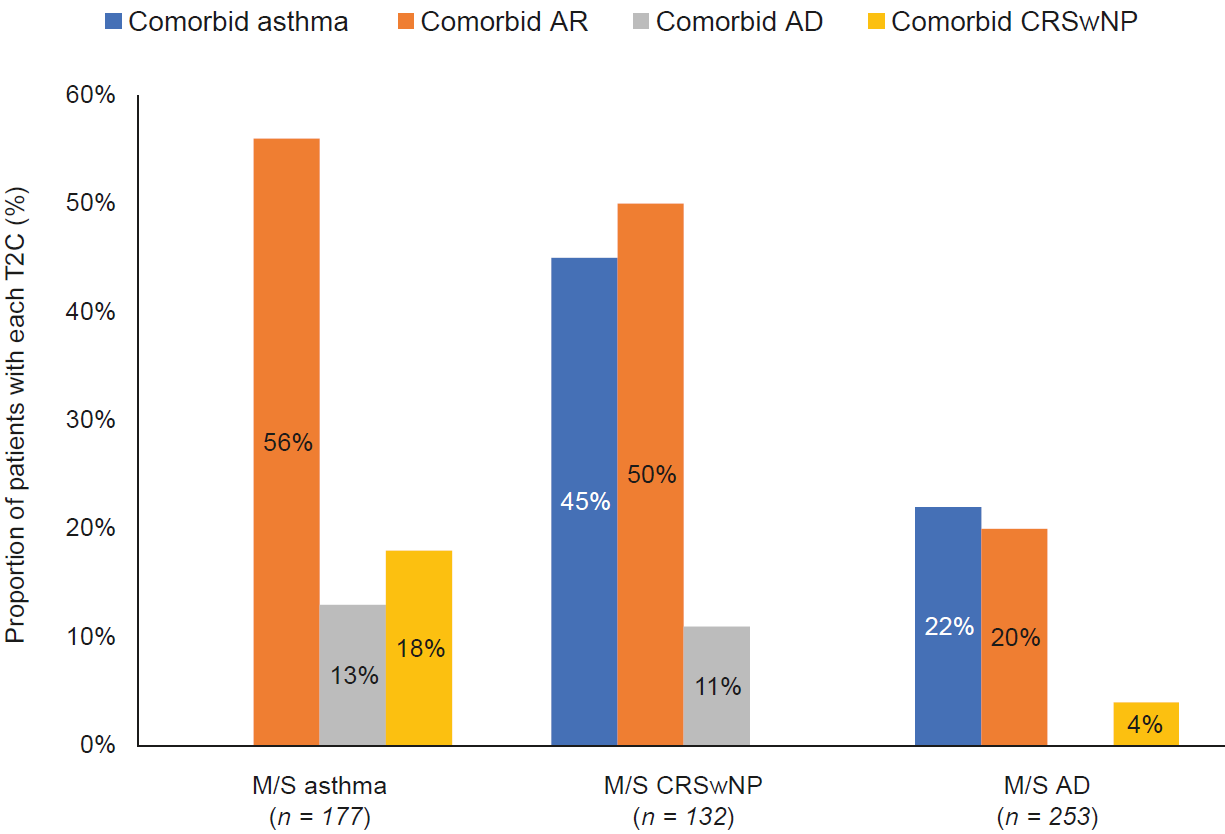 |
| **d** | 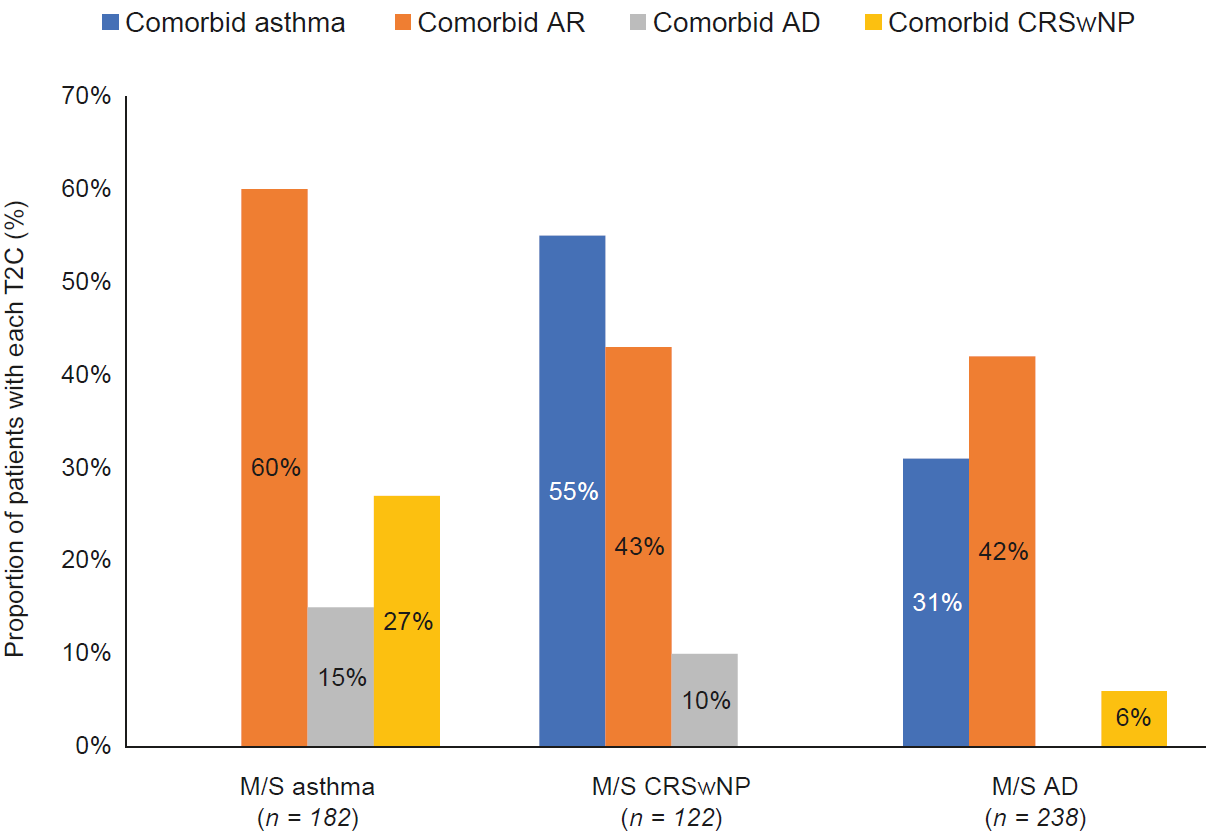 |
| **e** | 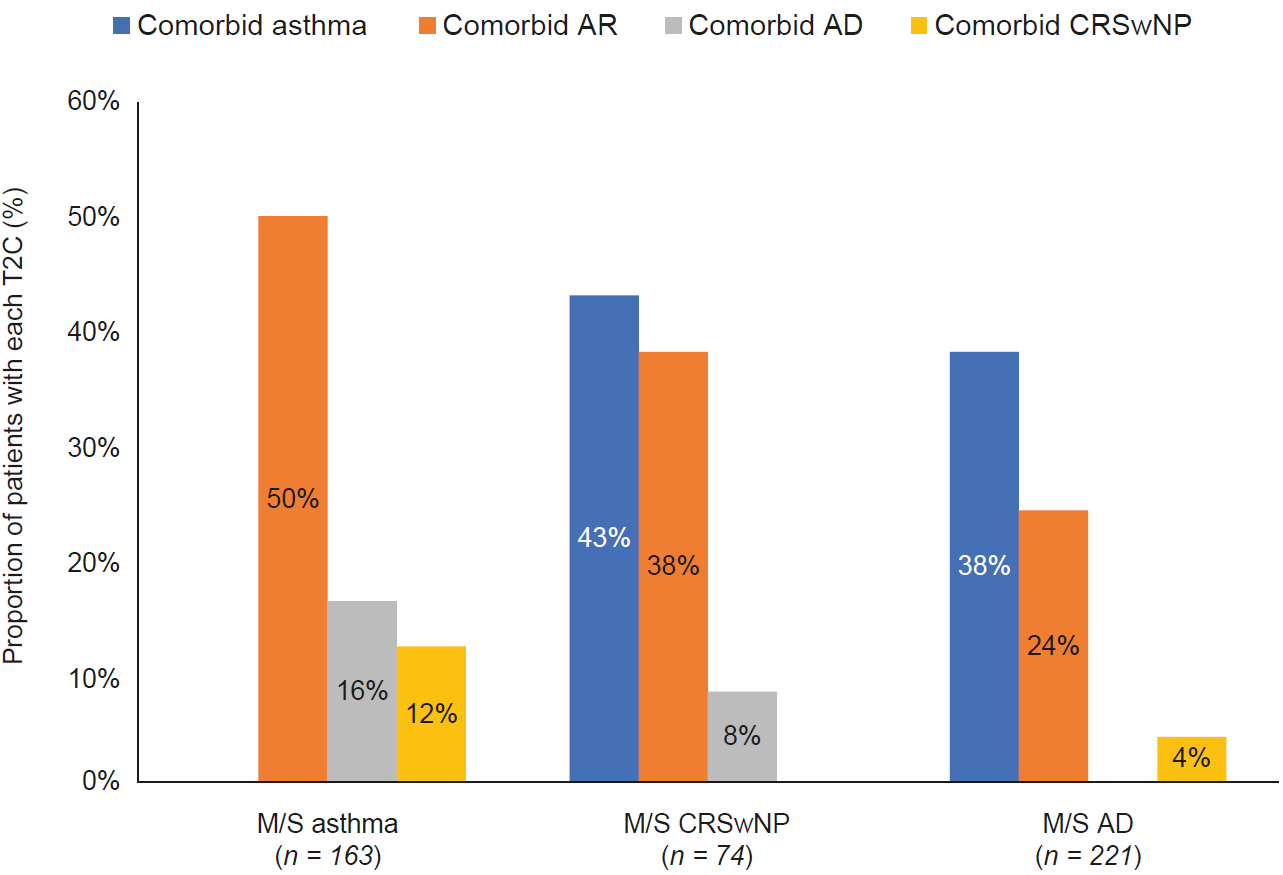 |

AD, atopic dermatitis; AR, allergic rhinitis; CRSwNP, chronic rhinosinusitis with nasal polyps; EUR5, France, Germany, Italy, Spain, United Kingdom; M/S, moderate-to-severe; T2C, co-existing type 2 inflammatory disease
